# Effects of Global Postural Reeducation versus Specific Therapeutic Neck Exercises on Pain, Disability, Postural Control, and Neuromuscular Efficiency in Women with Chronic Nonspecific Neck Pain: Study Protocol for a Randomized, Parallel, Clinical Trial

**DOI:** 10.3390/ijerph182010704

**Published:** 2021-10-12

**Authors:** Tânia Mendes-Fernandes, Ana Silvia Puente-González, Manuel Antonio Márquez-Vera, Carolina Vila-Chã, Roberto Méndez-Sánchez

**Affiliations:** 1 Centro EMA, 6300-537 Guarda, Portugal; taniafernandes_ft@hotmail.com; 2 Doctoral Programme in Health, Disability, Dependency and Welfare, University of Salamanca, 37007 Salamanca, Spain; amarvefisio@yahoo.es; 3 Department of Nursing and Physical Therapy, University of Salamanca, C/Donante de Sangre s/n, 37007 Salamanca, Spain; silviapugo@usal.es; 4 Institute of Biomedical Research of Salamanca (IBSAL), 37007 Salamanca, Spain; 5 Physiotherapy Unit, Universitary Hospital of Salamanca, 37007 Salamanca, Spain; 6 Polytechnic Institute of Guarda, 6300-559 Guarda, Portugal; cvilacha@ipg.pt; 7 Research Center in Sports Sciences, Health and Human Development (CIDESD), 5001-801 Vila Real, Portugal

**Keywords:** chronic neck pain, postural exercise, therapeutic exercise, global postural reeducation, disability, electromyography, postural control, neuromuscular efficiency

## Abstract

Background: Chronic nonspecific neck pain is the most frequent form of neck pain. It is more prevalent in women, and a costly public health issue. It is commonly associated with biomechanical, functional, proprioceptive, and postural impairments. The aim of this trial is to compare the effects of global postural exercises versus specific therapeutic exercises on neck pain, disability, mobility, pressure pain threshold, kinesiophobia, pain catastrophizing, postural control, and neuromuscular efficiency in women with chronic nonspecific neck pain. Methods and analysis: This study is a randomized, parallel-group and single blinded clinical trial. Sixty-two women with nonspecific chronic neck pain were recruited from the community of Guarda, Portugal, and randomly assigned to one of two intervention groups: (1) global postural reeducation (GPR group), (2) specific therapeutic exercises (STE group). The intervention was carried out over 4 weeks, with two sessions per week (eight sessions), and applied by a physiotherapist and paired with a daily individual at-home-exercise program. Primary outcomes are neck pain intensity and disability (Numerical Pain Rating Scale, Neck Disability Index). Secondary outcomes are cervical mobility and pressure pain threshold (CROM, algometry), attitude to pain (kinesiophobia, pain catastrophizing), standing postural control (Center of Pressure (COP) displacements), and neuromuscular efficiency (electromyography). There are four points of evaluation where the outcomes were assessed twice before the intervention, 1 week apart, and the two post-intervention assessments will be carried out after four and eight sessions. The objective was to increase scientific knowledge of different exercise modalities, such as global postural reeducation, in musculoskeletal disorders. Trial registration: ClínicalTrials.gov (NCT04402463), prospectively registered (data 22 May 2020).

## 1. Introduction

Nonspecific neck pain is an increasingly frequent musculoskeletal condition [1,2,3] affecting 22% to 70% of the population [4,5] that is more prevalent in women than in men [6]. Some patients will not experience complete resolution of pain and disability, which can become a more complex chronic pain syndrome [7]. When symptoms persist for more than 12 weeks, the condition acquires the value of chronicity and is denominated chronic nonspecific neck pain (CNSNP) [8]. This is associated with high costs for public health [9], and is becoming a socio-health problem [3].

The mechanisms underlying recurrence or persistence of neck pain may be associated with biomechanical, functional, proprioceptive, and postural changes [10,11,12,13,14,15]. However, the multidimensional nature of CNSNP can involve not only the sensory–motor component, but also psychosocial components, such as anxiety, depression, and fear and catastrophic thoughts in response to pain (kinesiophobia and pain catastrophizing) [4,16,17,18].

In postural control, the accurate functioning of the proprioceptive neck system is especially important in order to keep the joint position sense (JPS) and the movement control of the head and cervical spine intact [19]. Considering that neck muscles have a very high density of muscle spindles, especially in the suboccipital region [20], and that the cervical sensory afferences have strong influences on vestibular and visual systems to control postural stability [21,22], impaired neuromuscular function of the neck muscles can greatly influence postural control deficits [23].

Many studies have shown that people with cervical pain have abnormal contraction patterns of superficial and deep cervical flexor muscles [24,25,26,27,28], abnormal strength/endurance of deep cervical flexors (DCF) [29,30,31,32], impaired control and velocity of movement [33], proprioceptive and kinesthesic deficits [15,34,35,36,37], impaired postural control [13,15,22,23,38,39], decreased range of motion [3,15,40,41], low threshold for pressure pain, and general sensitization of the central nervous system [3,42,43,44,45].

Several non-pharmacologic treatments, such as manual therapy and therapeutic exercise, minimize neck pain and disability [46,47,48,49]. There are also other resources, such as pain neuroscience education, dry needling, shock waves, etc., that have shown beneficial effects on pain and disability [50,51,52,53]. It is always difficult to consider treatments with a single technique, and in this sense, some studies support the combination of different types of techniques or interventions for the treatment of neck pain [48,53]. Considering the evidence surrounding the variety of techniques in the treatment of neck pain, we wanted to consider of the reported beneficial effects of specific therapeutic exercises (STE), normally considered as tailored low-load exercises based on sensorimotor control, on neck pain, disability, dizziness, mobility, and muscle performance [46,47,49,54]. However, we also believe that the effects of different exercise modalities aimed at neck pain should be studied in depth.

Global postural reeducation (GPR) is a conservative treatment that we can consider as a global postural exercise method, and widely used in physical therapy clinical practice in many countries [55,56]. Some studies support the clinical effectiveness of GPR in treating patients with different musculoskeletal disorders and impairments [57,58,59,60,61,62,63], including cranio–cervico–mandibular complex [64,65,66,67,68,69,70,71,72]. The main aims of GPR are to reduce postural impairments and regain muscle symmetry and adequate posture through global active muscular stretching postures and joint decompressions, with breath control, motor control, contractions of antagonist muscles, and sensory integration exercises conducted by manual contacts in order to provide proprioceptive information to the patient [61,73,74,75,76,77]. These techniques are focused on reducing pain and improving body awareness and physical conditions, such as increased mobility, flexibility, muscle strength, and functional capacity [55,56,75,78,79], to finally obtain muscle balance and postural symmetry [61,67]. These findings must be considered with caution due to the heterogeneity of the results and the low quality of the studies that reported these results [55,56,59].

GPR is based on the concept that postural muscles are organized to act in concert with each other as “muscle chains” located anterior and posterior to the spine [55,61,67,74], and it has been hypothesized that some specific clinical presentations are caused by shortened muscles or “muscle chain retractions” [61,63,67]. Treatment with an active muscle stretching method exerts physiological effects not only at spinal but also at motor cortical level, increasing the amount of intracortical inhibition and/or reducing intracortical excitation, and induces a significant reduction of motoneuron excitation, which is caused by both pre- and postsynaptic mechanisms [62]. Moreover, according to the GPR basic principles of causality, globality, and muscle chain distribution [57,61,63,66,74], all of them containing the regional interdependence model of musculoskeletal dysfunction [80], the increased cortical inhibition following GPR was not limited to a specific muscle but tended to extend to segmentally related peripheral muscles [62].

However, there are few studies following the treatment of patients with chronic nonspecific neck pain with GPR, and no study has yet compared GPR with STE in NSNP and their effects on neuromuscular patterns or efficiency using an electromyographic assessment, as well as the correlations of this with pain, disability, standing postural control, and psychosocial components [55,56].

Considering that GPR is a therapeutic intervention with an approach based on an integrated idea of the muscular, it assesses and aims to act on the role and status of the functional groups of muscles responsible for posture and its alterations that form the muscle chains [55,56,66,67,81]. Most previous studies address pain and functionality as outcome variables [55,56], and very few address specific variables of postural balance [82], muscle efficiency with EMG [70], or psychosocial factors associated with pain such as kinesiophobia [67].

Additionally, although there is little previous evidence, results from previous studies with GPR have shown effects not only on symptom improvement, such as pain and functionality [55,56], but have also had effects on patient body awareness and proprioception associated with breathing and joint decompression [68,75,76]. Thus, in our study we anticipated that GPR would improve postural stability. Even though the interventions were not specifically designed to improve balance, they both aimed to enhance neuromuscular and sensorimotor control which could potentially enhance postural stability considering its neurophysiological mechanisms [19,21,22,23,83]. Moreover, this is the first study investigating the effects of GPR on the activity of cervical flexor muscles in patients with chronic neck pain by comparing it to an exercise mode that promotes coordination between the superficial and deep cervical muscles [84]. Only one preliminary study showed that repeated active postural exercise over 2 weeks decreased sternocleidomastoid muscle activity over some progressive stages of the cranio-cervical flexion test (CCFT) [85]. This active postural exercise involved assuming a sitting lumbo-pelvic neutral postural position with the addition of an active neck-lengthening maneuver. The authors suggested that this was accompanied by an improvement in the activation of the cervical DCF [84], and they proposed future studies with active postural exercises as an alternative treatment of neck pain [85]. Moreover, the majority of clinical trials studying GPR rated the method as a low to very low-quality treatment, which means that there is substantial uncertainty in their results [55,59].

Comprehensive interventions in postural and musculoskeletal disorders could make a significant contribution to treat and prevent recurrent episodes of pain, disability, psychosocial effects, and loss of work productivity [4,55,86,87,88,89]. Thus, it is vital to know what physiological functions different therapeutic exercises, including GPR, can and cannot address.

We considered the conclusions and indications of previous studies, based on the need for high-quality prospective clinical trials comparing GPR with effective treatment strategies for neck pain, such as manual therapy or therapeutic exercises [15,55,56,59]. Taking into account what has been described above, we considered it interesting to contribute more knowledge regarding the use in clinical practice of a wider variety of methods based on exercise and manual therapy. In our study we address the GPR, which due to the heterogeneity of the results of some studies, it presents some lack of evidence that we address in our study, on parameters in the application protocols and the effects on some outcome variables not sufficiently assessed in previous studies as patterns or neuromuscular efficiency, postural control and psychosocial factors, and also compare these effects with those caused by other therapies in this study factor (CNSNP).

Therefore, this randomized parallel clinical trial will aim to evaluate the benefits of GPR and STE, as two different exercise modalities, the former more dynamic [46,47,49,54], and the latter more static/postural [61,73,77], considering that both have mechanisms of action targeting symptomatology [46,47,55,56] and muscle efficiency [46,54,68]. So, pain, disability, postural control, neuromuscular function, and the correlations between them will be assessed in women with chronic non-specific cervical pain. The primary objective is to investigate the most effective and efficient intervention to improve neck pain intensity and disability. More concretely, we will evaluate if a global postural exercise method, such as GPR, or a specific therapeutic exercise has a superior effect. The secondary objective is to assess the effectiveness of the two interventions on cervical range of motion (CROM), pressure pain threshold (PPT) in the cervical region, kinesiophobia, pain catastrophizing, standing postural control, and neuromuscular efficiency of superficial cervical flexor muscles.

## 2. Materials and Methods

### 2.1. Study Design

This study is a randomized, parallel-group, and single blinded clinical trial. The protocol of the clinical trial received approval from the Ethics Committee of the University of Salamanca (ID: 458-2019), and shall be carried out in accordance with the Declaration of Helsinki. The informed consent is available in Spanish and Portuguese with the approved protocol. The clinical trial was registered at ClínicalTrials.gov (NCT04402463), prospectively registered (data 22 May 2020).

The study protocol conforms to the SPIRIT 2013 Statement (Standard Protocol Recommendations for Interventional Trials) [90], and the clinical trial conforms to the CONSORT 2010 Statement (Consolidated Standards of Reporting Trials) [91].

### 2.2. Participants, Recruitment and Sample Size Calculation

A sample of 62 women with CNSNP were recruited between 30 and 65 years old from the community in Guarda, Portugal. Participants were recruited through an online google form, with a questionnaire in Google Docs format, which was disseminated through social network postings (Facebook and Instagram) and the sending of institutional emails from Polytechnic Institute of Guarda to its workers. There were two physiotherapist assessors, who initially assessed participants that responded to the advertisements, and they were different from the physiotherapists who performed the interventions in both groups. In the first visit, the participants provided oral and written forms of all necessary information about the trial, including the study purpose and procedure. After voluntarily signing the informed consent statement, if through a detailed interview they met all inclusion, exclusion, and withdrawal criteria (Table 1), they enrolled into the trial to begin with an initial evaluation with the assessors.

The sample size calculation has been made based on the potential modification of the primary outcomes, neck pain intensity, and neck disability (NPRS and the NDI-PT) from baseline to final assessments. For repeated measurements of the estimated sample size, accepting an alpha risk of 0.05 and a beta risk of 0.2 in a two-sided test, 26 subjects were necessary in the first group and 26 in the second to recognize a statistically significant difference greater than or equal to 2 units (SD = 1.75) (NPRS) or 7 units (SD = 6) (NDI), and to detect small differences in standardized means between groups (Cohen’s d = 0.8), since the intervention with therapeutic exercise is known to have a beneficial effect and we want to assess whether the intervention with GPR has similar or even greater effects. Finally, considering a 15% loss rate during the study, and in order to end up with a sample of 52 subjects, based on the power analysis results, the final sample to be recruited was 62 subjects, 31 in each group. The sample size calculation was made with the software “PASS 15 (NCSS statistical software)” and “The R Project for Statistical Computing”.

### 2.3. Randomization and Blinding

After the baseline assessments, the physiotherapist that applied the interventions randomly allocated the participants to one of the intervention groups: the GPR group or the STE group. The randomization was undertaken by an independent assessor, with no other involvement in the trial, using a computerized randomization system (randomized.com; accessed date: 1 June 2020), and allocation concealment was guaranteed by sequentially numbered, opaque, sealed envelopes. Due to the nature of the intervention itself, the participating subjects were not blinded to the intervention, however, they did not know what type of exercise they received, having been informed only that they will receive an effective exercise treatment. To minimize contamination between groups, the assessors performing the study measurements of all outcome variables and the statistical analysis were blinded. The assessors did not know the group to which the subjects belonged in each of the evaluations, and the statistician performed the statistical analysis once all the variables were coded.

### 2.4. Procedures

**Figure 1:**
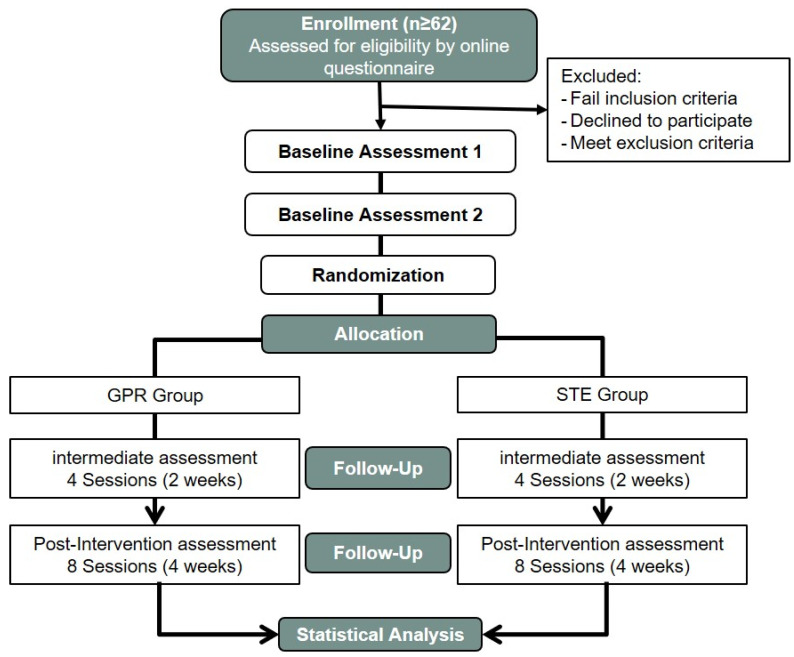
Flow chart of participants in the study. GPR: Global Postural Reeducation; STE: Specific Therapeutic Exercises.

Following the parameters of usual clinical practice and considering previous studies of GPR, according to some systematic reviews [55,92], and the protocols of STE in neck pain [54,93,94], the interventions were provided in eight sessions over a 4-week intervention period (two visits per week) accompanied by an individual daily at-home exercise program [64]. Baseline and follow-up assessments were conducted at the Polytechnic Institute of Guarda. There were four assessment points, and the time factor was considered in the statistical analysis with four levels. Initially, a test–retest reliability analysis was conducted on all participants to verify the variability between days. To achieve this, we applied two pre-intervention assessments, 1 week apart. After the second pre-intervention assessment, participants started the interventions in each group, and they will be assessed again in an intermediate evaluation after 2 weeks and four sessions of treatment (assessing only neck pain intensity, CROM, and cervical PPT outcomes) and in one last evaluation at the end of the intervention, 4 weeks and eight sessions of treatment (assessing all outcomes, such as in pre-intervention assessments). All data collected were confidential and private, ensuring anonymity of respondents. The participants were advised not to use other forms of treatment during the trial (pharmacologic or non-pharmacologic treatment). See the flow chart in Figure 1.

### 2.5. Interventions

The interventions were conducted by an experienced physiotherapist with more than 7 years in the treatment of the musculoskeletal disorders with these interventions.

The interventions in both groups commenced after the second evaluation, which we considered the baseline assessment. The interventions in both groups were performed for 4 weeks, and they consisted of a combination of treatment sessions carried out by a physiotherapist and exercises to be performed at home. The physiotherapist performed two sessions per week [64] with the participants in each group, and participants were asked to practice their prescribed exercise program at home, depending the group, once daily for 4 weeks, and to complete an exercise diary to monitor compliance and record adverse events. Each session of treatment lasted approximately 35 min for the STE group and 45 min for the GPR group, according to clinical practice and previous studies [54,55,92,93,94]. The physical therapist taught and showed a detailed home exercise program for all participants, where the physiotherapist provided easy and simple home exercises without devices. Participants in GPR group performed at home one lying posture and the STE group prescribed active movements and stretches of the neck muscles (Table 2).

**Figure 2:**
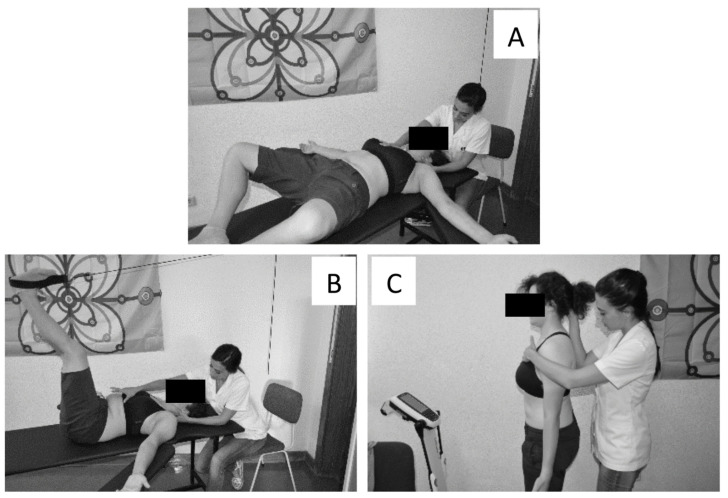
The 3 positions of the Global Postural Reeducation intervention. (**A**) Lying posture to stretches the anterior muscle chain; (**B**) lying posture to stretches the posterior muscle chain; (**C**) standing posture.

Participants assigned to the GPR group performed global postural exercises consisting of three positions, following the protocol of Lozano-Quijada C. et al. [82] (Figure 2). Initially, without gravity load, a lying posture was applied to the anterior muscle chain for approximately 15 min, then another lying posture to the posterior muscle chain for other 15 min, and finally the patients worked in a standing posture for postural integration under a gravity load for 5 min. Between each posture there was a short resting time.

**Figure 3:**
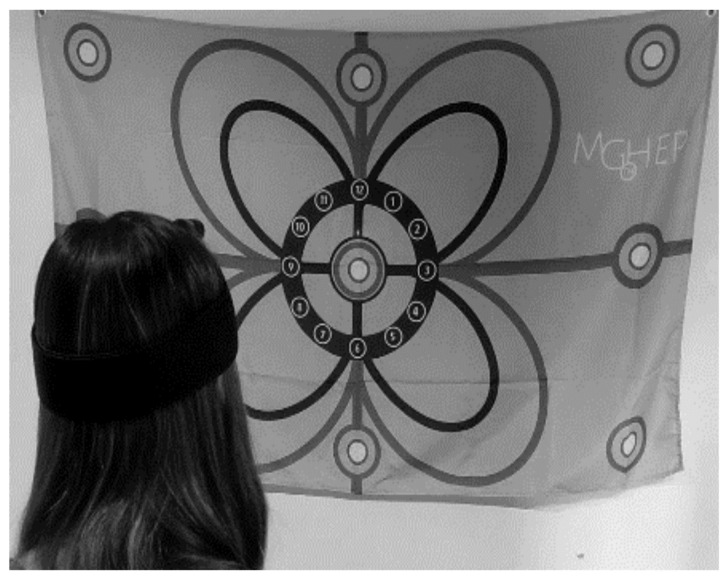
Specific Therapeutic Exercise intervention. Exercises with visual feedback with a laser sensor (Motion Guidance).

Participants assigned to the STE group performed specific therapeutic exercises for motor control, divided into three phases, following the protocol of Jull G. et al. [54,93] and Sremakaew M. et al. [94]. Each of the phases had two exercises with visual feedback with a laser sensor (motion guidance: a laser pointer rehab tool that adds visual feedback for motor control during exercise in physical therapy) (Figure 3).

In both interventions, all exercises were performed following a progression in intensity. The physiotherapist tried to ensure that the patients performed all the exercises as correctly as possible with the appropriate and expected intensity, although it was necessary to be careful and always consider possible exercise adaptations to respect the individuality, as a principle of GPR, of each subject (such duration of the pause between exercises, changes in manual contacts, time applied to each posture, or type of breathing of GPR).

The exercise programs for both interventions are described in Table 3. In addition, details of the exercise progression for each group are presented in Table 4 and Table 5.

In GPR the process of progression will depend a lot on the individuality of each participant. This means that we increased the stretch while the participant was able to maintain all postural corrections without compensation.

### 2.6. Outcome Variables

In the first pre-intervention assessment, all variables were measured, including the sociodemographic variables. Later, all outcome variables were measured in the second pre-intervention assessment (baseline outcomes) and in the final assessment after the completed interventions (eight sessions in each group) (Table 6). As indicated above, in the intermediate evaluation (after four sessions of treatment) only the neck pain intensity, CROM, and PPT outcomes will be assessed (Figure 1).

Personal and sociodemographic variables:

- Age (years);
- Sex (male or female);
- Weight (kg); Height (m); Body mass index (kg/m^2^);
- Time feeling neck pain (years, months and weeks);
- Education level (no studies, basic education, secondary school, or superior studies);
- Marital status (single, married, separated, divorced, or widowed);
- Employment status.

#### 2.6.1. Primary Outcome Variables

The primary measured outcomes of the study will be the change within groups and the differences between groups of the neck pain intensity and the neck disability at the assessment times.

- Neck pain intensity

Neck pain intensity will be measured using a numerical pain rating scale (NPRS). This is an 11-point scale ranging from 0, which represents one pain extreme (e.g., “no pain”), to 10, which represents the other pain extreme (e.g., “pain as bad as you can imagine” and “worst pain imaginable”) [95]. The subject is instructed to select the number that best represents the mean of their pain in the last week. Evidence suggests that the perception of pain intensity in subjects with chronic pain must be analyzed by NPRS, and it has been demonstrated to be a valid and reliable scale [96,97]. NPRS has a high test–retest reliability and responsiveness when applied intra-observer reliability of (r = 0.76) in patients with mechanical neck pain [97] and in both literate and illiterate patients with rheumatoid arthritis (r = 0.96 and 0.95) [98]. NPRS also has a high validity with visual analogue scale (VAS) in patients with rheumatic and other chronic pain conditions (pain > 6 months) (correlations range from 0.86 to 0.95) [95], and a high convergent validity with VAS and verbal rating scale (VRS) (r = 0.82–0.92) [99]. Chronic pain patients prefer the NPRS over other measures of pain intensity due to its comprehensibility and ease of completion [99,100].

- Neck Disability

We will measure the neck disability associated with pain using the Portuguese version of the neck disability index (NDI–PT), which is valid and reliable with excellent internal consistency (Cronbach’s alpha = 0.95). NDI–PT has a high test–retest reliability with an ICC = 0.91 [101,102], and NDI also has a highly convergent validity with a variety of pain scales (r = 0.40–0.71) [101]. NDI–PT is a patient-completed, condition-specific functional status questionnaire with 10 items including pain, personal care, lifting, reading, headaches, concentration, work, driving, sleeping, and recreation. Each section is rated on a scale of 0 to 5, where 0 means “painless” and 5 means “worst pain imaginable.” The points obtained are added to a total score between 0 and 50. The NDI-PT scores are presented as a percentage of the maximum score, 0–8% without disability; 10–28% mild; 30–48% moderate; 50–64% serious, and 70–100% complete disability [103,104].

#### 2.6.2. Secondary Outcome Variables

The secondary outcomes in the study will be the change within groups and the differences between groups in standing postural control, the neuromuscular efficiency in superficial cervical flexor muscles, the CROM and PPT in the neck, and the attitude and thoughts in response to pain.

- Standing Postural Control

Postural assessment was conducted with the subjects standing on a force platform (Kistler, model 9260AA6, Winterthur, Switzerland) following the recommendations of the systematic review published by Ruhe A. et al. to achieve acceptable to good reliability for most center of pressure (COP) parameters [105,106]. Data were sampled at 1000 Hz over 40 s in a standing position, and different parameters of COP excursions were analyzed (total sway area, antero-posterior and medio-lateral COP displacement, mean COP velocity, and antero-posterior and medio-lateral COP velocity) [105,106,107]. Participants were asked to stand upright with their arms by their sides and barefoot on the top of the platform in four different positions: (1) narrow stance (feet together) with open eyes, (2) narrow stance with closed eyes, (3) narrow stance on a spongy surface, with open eyes, (4) narrow stance on a spongy surface, with closed eyes. Participants will be required to maintain each position twice, and the second time was considered for the study [107,108].

- Electromyography (EMG)—Cranio-cervical Flexion Test (CCFT)

**Figure 4:**
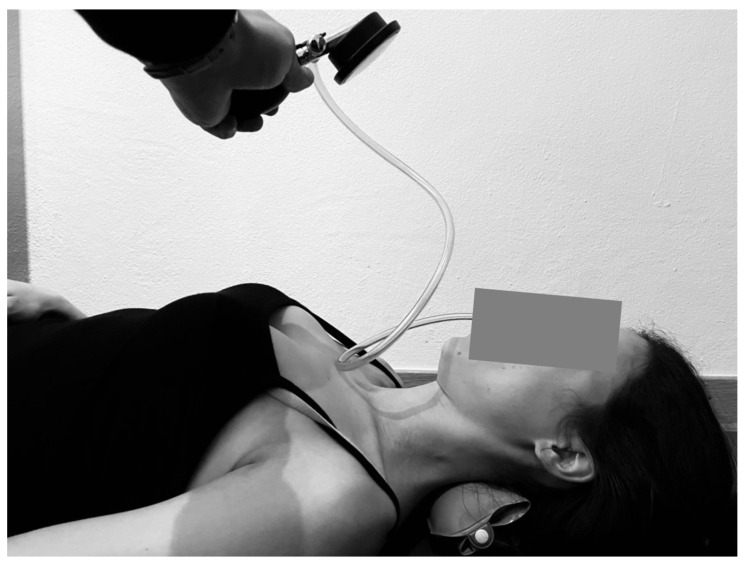
Cranio-cervical flexion test (CCFT) with visual feedback (Stabilizer).

To investigate the amplitude of the muscle activation, bipolar surface EMG signals were detected from the sternal head of the sternocleidomastoid (SCM) and anterior scalene (AS) muscles bilaterally during the CCFT in accordance with established protocols [83,109,110]. Pairs of Ag–AgCl electrodes (Ambu Neuroline; conductive area 28 mm^2^) were positioned 20 mm apart over the SCM and AS following skin preparation and using guidelines for electrode placement [111]. EMG signals were amplified as bipolar derivations (EMG amplifier; LISiN-OT Bioelettronica, Torino, Italy), band-pass filtered (−3 dB bandwidth, 10–500 Hz), sampled at a rate of 2048 samples/s, and converted to digital data by a 12-bit analog-to-digital (A/D) converter board. The reference electrode (wet wrist strap, WS1, LISiN-OT Bioelettronica) was placed around the wrist, over the ulna and radius styloid processes. Subjects were comfortably positioned in a supine position, lying with the head and neck in a mid-position, and were instructed to perform a cranio-cervical flexion action (anatomical action of the DCF). The task consisted of five incremental movements of increasing cranio-cervical flexion ranges of motion. Performance was guided by visual feedback from an air-filled pressure sensor (Stabilizer, Chattanooga Group Inc., Austin, TX, USA), which will be placed in the suboccipital region, behind the subject’s neck, and inflated to a baseline pressure of 20 mmHg (Figure 4) [24,83,107]. The pressure sensor monitors the slight flattening of the neck, which occurs, with the contraction of the longus capitis and longus colli muscles [112]. During the test, subjects were required to perform the gentle nodding motions of cranio-cervical flexion, progressing in range to increase the pressure by five incremental levels, with each increment representing 2 mmHg (22–30 mmHg). Subjects practiced targeting the five test levels between 22 and 30 mmHg in two practice trials before the electrodes were applied. Following the application of electrodes, participants will perform a standardized maneuver for EMG normalization (reference voluntary contraction) [24]. This reference voluntary contraction involved a head lift (cervical and cranio-cervical flexion) just clear of the bed, which was maintained for 10 s, during which EMG data are recorded [24]. A 1-min rest period was given before participants perform the experimental CCFT and EMG data are recorded. The experimental CCFT will include all five stages of the test (22–30 mmHg), with participants instructed to maintain steady pressure on each stage target for 10 s, and rest for 30 s between stages. For each of the incremental pressure levels, recording of EMG data commenced when the assessor observes that the participant has reached the pressure target. A consistent starting point for each level was attained by ensuring the participant has returned to the neutral head/neck position, which corresponds to the pressure level reading of 20 mmHg. The average rectified value (ARV) was computed offline from the EMG signals in intervals of 1s. The ARV values were then averaged and normalized with respect to the ARV computed from the reference voluntary contraction and expressed as a percentage [113].

- Cervical mobility

A range of motion instrument was used to determine cervical spine movements (CROM Deluxe, Performance Attainment Associates, Roseville, Minnesota, United States). This device consists of an instrument placed over the head that determines the degree of cervical flexion, extension, inclination, and rotation. The evaluation was conducted in a sitting position, and the average of three measures for each direction considered. The CROM has been shown to have excellent test–retest reliability (ICCs = 0.89–0.98) and high validity (r with Fastrak motion analysis system = 0.93–0.98) [114].

- Cervical Pressure Pain Threshold (PPT)

The PPT was recorded using a calibrated digital algometer (Force Ten™ -Model FDX; Wagner, Greenwich, CT, USA) with a round tip surface area of 1 cm^2^. This device quantified the threshold of pain to pressure (kgf) referenced by subjects at evaluation points [107]. The assessor gradually increased the pressure over these points bilaterally, in the posterior cervical spine at the level of the second (C2) and sixth (C6) vertebrae (prone position), upper trapezius muscle (sitting position), and over the tibialis anterior muscle (supine position) [115,116,117,118]. The test was repeated three times at each point and the average was considered, until the patient indicates when the pain or discomfort appears [119]. The algometry measurement of PPT has been shown to have excellent test–retest reliability (ICC = 0.91; 95% CI 0.82–0.97) [119].

- Attitude to pain

To evaluate attitude and thoughts in response to pain, we will assess kinesiophobia and pain catastrophizing:

1. Tampa Scale of Kinesiophobia (TSK)

Kinesiophobia will be measured using the Portuguese version of the 13-item Tampa Scale of Kinesiophobia (TSK-13-PT) that evaluates the fear of movement and reinjury [120]. This questionnaire contains 13 questions, each of which are scored from 1 to 4. Total scores range from 13 to 52, with a higher score indicating higher levels of kinesiophobia, and it can be categorized into four intensity ranges: “subclinical” (13–22); “mild” (23–32); “moderate” (33–42); and “severe” (43–52) [121]. The Cronbach alpha of 0.82 indicated good internal consistency of the TSK-13-PT total score, and the 1-week ICC of 0.99 indicated exceptional test–retest reliability [120].

2. Pain Catastrophizing Scale (PCS)

To evaluate the tendency to magnify the threat value of a pain stimulus and to feel helpless in the presence of pain, we will use the Brazilian Portuguese version of the PCS (BP-PCS) [122]. This scale is composed of 13 statements, and participants are prompted to describe the frequency with which they experience different thoughts and feelings associated with pain using 5-point scales with the extreme points (0) not at all and (4) all the time. The 13 statements are grouped into three subscales: rumination (4 items), magnification (3 items), and helplessness (6 items). Total score ranges from 0 to 52 and higher scores are indicative of higher catastrophic thinking [123,124]. The BP-PCS demonstrated good internal consistency (Cronbach α = 0.91) for the total score (Cronbach’s α = 0.91), and also good internal consistency with 0.93 (helplessness), 0.88 (magnification), and 0.86 (rumination) for the respective subdomains [122]. The item-total correlation coefficients ranged from 0.91 to 0.94 [122].

### 2.7. Patient and Public Involvement

Patients and other members of the public were involved in the design and management of the clinical trial and will be involved in conducting the trial. Firstly, we organized a public meeting for workers of the Polytechnic Institute of Guarda and people of the community of Guarda interested in the purpose of the clinical trial, where they could contribute with opinions and inputs for the study. Once the trial has been published, we also intend to disseminate the main results to trial participants and will seek to develop an appropriate method of dissemination to the general population.

### 2.8. Statistical Analysis

Data were analyzed by using the IBM-SPSS software package (version 23.0). Descriptive data analysis was reported by groups as means ± standard deviation for numeric variables and as frequencies and percentages for categorical variables. In the graphical representation, bar graphs by groups were used for numeric variables, and pie charts were used to represent categorical variables. The main change from baseline to 4 weeks of treatment will be calculated using an intention-to-treat analysis for each outcome measure.

Two-way repeated measures ANOVA were performed. Group factors, as age range, were considered as categorical variables in the statistical model, such as potential covariates, and the Time factor will be used to take into account the two pre-intervention (baseline) and the intermediate and final data in the intervention (2 and 4 weeks of treatment). The interaction between the two factors on the dependent variables was analyzed. When the interaction was statically significant, follow-up tests (post-hoc Sidak test) were performed to determine how the within-participant factors affected the dependent variables, and it could be used as a covariate in the model. The post-hoc Sidak test was calculated to determine which pairs of means have significant differences.

Correlations with Pearson’s r coefficient were determined to analyze whether the values of two or more numeric variables change in conjunction or are related at some time point of the study.

The level of significance for the statistical tests were set at *p* ≤ 0.05 with a confidence interval of 95%.

In order to focus on clinical relevance or clinically worthwhile effects, and assess the magnitude of the change in the result variables, the effect size of both treatments for the group × time repeated-measures ANOVA model were calculated as the partial eta squared (ŋp^2^) when significant, considering 0.01 small, 0.06 medium, and more than 0.14 large effect sizes, and we could use the Cohen d for any pairwise comparisons, when applicable.

### 2.9. Ethics and Dissemination

This study has been approved by the Ethics Committee of the University of Salamanca (ID:458-2019), and shall be carried out in accordance with the Declaration of Helsinki. The informed consent is written and accessible in Spanish and Portuguese with the approved protocol, and all participants will give consent to participate prior to any study-related procedures. Adverse effects from the treatment are not expected to occur, but if they do, the Public Health System will cover all patients, and the participants will be informed of this. The results will be presented at international conferences and published in peer-reviewed journals. Negative, positive, as well as inconclusive results will be published.

## 3. Discussion

Neck pain is a common problem, and it has been estimated that 67% of the population will suffer from it at some moment in their life [50]. The most frequent presentation of neck pain is nonspecific or mechanical neck pain [3] that turns into a real health problem with high costs when it becomes chronic [7,9].

Many physiotherapy techniques have shown beneficial effects in patients with CNSNP. Therapeutic exercise is considered as one of the most important, either applied alone or with other interventions such as manual therapy.

As in other musculoskeletal disorders, in CNSNP it is necessary to determine what type of exercise, what dose, and what intensity or duration can be the most efficient treatment. Not only must pain be considered the main symptom, but treatments must be focused on functional and psychosocial disorders that can contribute to the perpetuation of pain and other complications that are not spontaneously reversible.

This clinical trial intended to show the treatment of CNSNP with GPR as a global postural method, with global active muscular stretching postures, motor control, and contractions in order to provide proprioceptive information to achieve correct postural integration and therefore good postural control, as an alternative to STE of the neck.

This protocol presented a detailed description of a randomized parallel trial designed to analyze the results in terms of pain, disability, neuromuscular efficiency, postural control, CROM, PPT, kinesiophobia, and pain catastrophizing with two types of treatments for non-specific chronic neck pain.

The aim of this trial was to contribute to increased scientific knowledge of the effect of GPR, as a complex and global method that combine manual and active interventions, in musculoskeletal disorders on a great variety of outcome variables and initiate new lines of future research. Data will be published after the study is completed.

## Figures and Tables

**Table 1:** Inclusion, exclusion, and withdrawal criteria.

Inclusion Criteria
Aged between 30 and 65 years Female Persistent cervical pain for more than 12 weeks at the time of inclusion Pain intensity equal to or greater than 2 points in the numerical pain rating scale (NPRS)
**Exclusion Criteria**
Specific cause of cervical pain with previous medical diagnosis (e.g., traumatic, rheumatic or systemic pathology) Central or peripheral neurological signs Cognitive decline Cervical surgery Known or suspected vestibular pathology, dizziness, sensory nerve pathways or vascular disorders (e.g., migraine, hypertension) Musculoskeletal and neurological conditions that could affect balance Taking any pharmacologic treatment Received physiotherapy treatment for their neck pain in the last 3 months
**Withdrawal Criteria**
To use other forms of treatment during the trial (pharmacologic or non-pharmacologic treatment)

**Table 2:** Description of home exercises.

GPR group	The subject practices an autoposture in lying position on the floor, it should be performed without pain and keeping the same breathing as what perform with the physical therapist in sessions. In supine position, with back stretched, flexion of hips and knees and closed legs, the participant exhale deeply. After some breathings the subject move the hips in a posterior pelvic tilt position and stretch the neck with a cranio-cervical flexion. The subject maintains the position with breathing in different angles of the arms in abduction.The subject needs to repeat the exercises during 15 min every day except in the days of presential session.
STE group	The subject performs neck exercises sitting in a chair (Flexion, extension, rotation and inclination slow movements and global stretches of the main muscle groups of the neck with the help of the hands (flexors, extensors and lateroflexors). 10 repetitions each movement and 20 s for each stretch. The execution of all exercises should take about 15 min, and each exercise should be performed without causing pain while maintaining a quiet breathing.

**Table 3:** Description of exercise programs used in the interventions.

Modality	Description
Postural Global Reeducation [82]	At each session, patients will maintain two different lying postures and one standing (Figure 2): Stretching of the anterior muscular chain: patients will be positioned in the supine position and initiate flexion from the lower limbs and end with the extension of both hips and knees; Stretching of the posterior muscular chain: in a supine position, the progression includes hip flexion (90 degrees) and knee extension. For both postures, manual traction will be applied to both lumbar and cervical areas, and isometric contractions of the stiff muscles will be elicited to induce post-isometric relaxation. Integration Standing in the center: The final part of the treatment will be aimed to facilitate the integration of the postural correction into daily functional activities. During the global stretching session, care will be taken to avoid postural compensation (due to tension increase in response to muscular tightness) on specific body segments, and patients will maintain free breathing, with no breath holding.
Specific therapeutic exercises [54,93,94]	Cervical flexors 1. Train craniocervical flexors (CCF) activation and holding capacity. 2. Train the interaction of deep and superficial cervical flexors in movement patterning and functional tasks. 3. Train co-contraction of the deep cervical flexors and extensors. 4. Train strength and endurance of the cervical flexors.
	Cervical extensors 1. Train craniocervical extensors and rotators with the cervical spine in a neutral position. 2. Train cervical extension to bias the cervical extensors (extend cervical spine keeping the craniocervical region in a neutral position). 3. Train strength and endurance.
	Axioscapular muscles 1. Train scapular muscles, in particular the upper/middle/lower trapezius and serratus anterior, in both open and closed chain positions, with and without load and movement of the upper limb. 2. Train correct scapular posture.
	Postural correction exercise 1. Train a neutral spinal posture. 2. Train scapulothoracic and cervical postures.
	Sensorimotor exercises with a visual feedback rehab laser (Figure 3) Using a laser pointer mounted onto a lightweight headband, participants practice: Relocation of the head back to a neutral posture or to predetermined points in range. The exercise will be progressed by closing the eyes and by changing directions and ranges of movement. Movements of the head to points in different directions (horizontal and vertical lines and circles) of the different designs of Motion Guidance. The exercises will be progressed by increasing speed and tracing more intricate patterns such as a figure eight, zig-zag, or a butterfly form.

**Table 4:** Details of the progression of the global postural exercises (global postural reeducation).

Global Postural Exercises (GPR) [82]			
Phases	Description		
**1**	**Lying posture—without gravity load** In order to achieve and maintain postural balance, specific exercises in the lying position will be used. These exercises involve a precise use of contractions, stretch reflexes, light and controlled manual traction, and sustained elongations.		
**A**	**Stretching of the anterior muscular chain**	**Time/mode**	**Progression**
	Supine position; physiotherapist begins with specific focus on breathing according to the need of each patient and soft cervical traction (stretching muscles involved in breathing: scalenes, minor pectoral, intercostal, and diaphragm). Supine position with upper limbs at 45° of abduction and flexed, abducted, and laterally rotated hips, with the soles of the feet touching each other to stretch the anterior muscle chain (diaphragm, pectoralis minor, scalene, sternocleidomastoid, intercostalis, iliopsoas, arm flexors, forearm pronators, and hand flexors). The pelvis will be kept in neutral position with an initial traction of the sacrum, while the lumbar spine remains stabilized. The physiotherapist will stretch the superior shoulder muscle chain (upper trapezius, elevator scapulae) with upper limbs into adduction (to adduction from 45° to 0°), emphasizing breathing and cervical traction. The correct alignment of the patient will be accomplished throughout stretching of the thigh muscles and followed by repositioning of the segments/joint, through soft isometric contractions in more elongated positions to induce post isometric relaxation, in order to improve postural alignment awareness of that posture. The maintenance of alignment during posture will be achieved by verbal commands and manual contact of the therapist, guarantying the active engagement of patient to reach the correct posture. At the same time, gradually, the lower limbs will be extended as much as possible while maintaining the corrections.	4 min (stretching) 12 min (contractions, stretch reflexes, light and controlled manual tractions, and sustained elongations to realign posture until finishing the posture with extended limbs)	Manual traction will be applied to the sacrum and to the occiput to align the curves of the spinal column. Progressive abduction and lateral rotation of the hips, then extension, adduction, and neutral rotation. Progressive adduction of the shoulder joints. Deep rhythmic expiratory breathing throughout.
**B**	**Stretching of the posterior muscular chain**	**Time/mode**	**Progression**
	In order to stretch the posterior muscle chain (upper trapezius, levator scapulae, suboccipitalis, erector spinae, gluteus maximus, ischiotibials, triceps surae, and foot intrinsic muscles), the patient will lay in the supine position with the occipital, lumbar, and sacral spine stabilized, with the lower limbs at 90° hip flexion, and perform gradual knee extensions. In both of the postures the correct alignment of the patient will be accomplished throughout stretching of the thigh muscles and followed by a repositioning of the segments/joint, through soft isometric contractions in more elongated positions to induce post isometric relaxation, in order to improve postural alignment awareness of that posture. Contractions, stretch reflexes, light and controlled manual tractions, and sustained elongations to realign posture and finish with extended limbs. The maintenance of alignment during posture will be achieved by verbal commands and manual contact of the therapist, guarantying the active engagement of patient to reach the correct posture. At the same time, gradually, the lower limbs will be extended as much as possible while maintaining the corrections.	12 min (contractions, stretch reflexes, light and controlled manual tractions, and sustained elongations to realign posture until finishing the posture with extended limbs)	Manual traction will be applied to the sacrum and to the occiput to align the curves of the spinal column. Progressive increase of flexion, adduction, and neutral rotation of the hips, knee extension, and dorsiflexion of the ankles. Progressive adduction of the shoulder joints. Deep rhythmic expiratory breathing throughout.
**2**	**Standing posture—integration under gravity load**		
**C**	**Standing in the center**		
	With the participant standing with an open hip angle and slightly flexed knees, the physiotherapist will make final corrections for postural integration for the stretching while the participant extends the knees, maintaining the correct posture of the spine and upper and lower limbs.	5 min	Progressive extension, adduction, and neutral rotation of the hips. Manual traction will be applied to the occiput throughout the feet and toes in a normal alignment with the floor throughout. Progressive adduction with neutral rotation of the shoulder joints. Deep rhythmic expiratory breathing throughout.

**Table 5:** Details of the specific therapeutic exercise progression.

Specific Therapeutic Exercises [54,93,94]			
Exercise	Level 1	Level 2	Level 3
Cervical flexor	Re-education of CCF movement pattern Supine, knees bent - Gentle and controlled nodding action facilitated with eye movementHolding capacity Supine, knees bent - Repeated and sustained CCF progressing from 22 to 30 mmHg10 repetitions	Interaction between the deep/superficial cervical flexors Sitting - Controlled head movement through range of extension and return to neutral 10 repetitions Co-contraction of the deep cervical flexors/extensors Sitting - Isometric cervical rotation facilitated with eye movement (left/right sides) 5 s holds × 5 repetitions	Strength/endurance of the cervical flexors Sitting - Isometric CCF in a range of cervical extension - Lifting the head off the wall (with the chair up to 30 cm away from the wall) Supine - Lifting the head off a pillow (2, 1, then 0 pillows as per participant’s capacity) 10 s holds × 10 repetitions
Cervical extensor	Re-education of extension movement pattern Prone on elbows/four-point kneeling positions - Craniocervical extension - Craniocervical rotation (<40°) - Cervical extension while keeping the craniocervical region in a neutral position 3 sets of 5 repetitions	Co-contraction of the deep cervical flexors/extensors Sitting - Isometric cervical rotation facilitated with eye movement (left/right sides) 5 s holds × 5 repetitions	Strength/endurance of the cervical extensors Prone on elbows/four-point kneeling positions - Isometric hold in range of cervical extension - Addition of progressive load (light weights attached to head) as per patient’s capacity 10 s holds × 10 repetitions
Axioscapular control	Re-education of scapular movement control Side lying with arm elevated 140°/sitting - Passive repositioning of the scapular - Active repositioning of the scapular Holding capacity Side lying with arm elevated 140°/sitting 10 repetitions - Active repositioning the scapular posture and isometric hold 10 s holds × 10 repetitions	Axioscapular muscle control Sitting - Arm movement without load (external rotation/abduction/flexion < 30°) - Arm movement without load throughout range 10 repetitions Prone on elbows/four-point kneeling position - Thoracic lift (serratus anterior) and isometric hold 5 s holds × 5 repetitions	Strength/endurance of axioscapular muscles Sitting - Arm movement with load (external rotation/abduction/flexion < 30°) - Arm movement with load throughout the range 10 repetitions Prone - Lift the shoulder off the bed and hold without arm load - Lift the shoulder off the bed and hold with arm load 10 s holds × 10 repetitions
Postural correction	Correction of spinal posture Sitting - Active upright sitting initiated with lumbo-pelvic movement 10 s holds × 10 repetitions	Correction of spinal posture and scapular orientation Sitting - Actively positioning the scapular in a neutral posture while maintaining spinal posture 10 s holds × 10 repetitions	Spinal and scapular correction plus occipital lift Sitting - Actively lengthen the back of the neck while maintaining spinal and scapular posture 10 s holds × 10 repetitions
Motion Guidance (visual laser feedback)	Correction of the head position—Retropulsion of the head—keeping laser in the center	Cervical JPS with eyes open 5 times on each side (horizontal, vertical, and diagonal)	Draw the entire flower sitting and standing 2 times on each side
	Trunk head coordination—Rotate trunk keeping head and looking stability	Cervical movement sense: Draw the flower on the blue lines, 2 times for each side	Slow and fast speed training drawing the flower
	Cervical movement sense: overlapping vertical and horizontal lines with laser 2 min each	Laser head retropulse keeps 10× in the center of the foot 2 min each	Cervical JPS. Joint replacement with eyes closed 5 times on each side (horizontal, vertical and diagonal 2 min each

**Table 6:** Summary of outcome variables.

Primary Outcome Variables	Data Collection Tools
Neck Pain intensity	NPRS (scale range 0–10)
Neck Disability	NDI-PT (scale range 0–50)
**Secondary Outcome Variables**	**Data Collection Tools**
Standing Postural Control	Stabilometry (COP displacement parameters)
Neuromuscular efficiency	Electromyography—(CCFT)
Cervical mobility	CROM (degree)
Cervical Pressure Pain Threshold	Digital algometer (kgf)
Attitude to Pain	TSK-13-PT (scale range 13–52)
	PCS-PT (scale range 0–52)

NPRS: Numerical Pain Rating Scale; NDI-PT: Neck Disability index-Portuguese version; COP: Centre of Pressure; CCFT: Cranio-cervical flexion test; CROM: Cervical Range of Motion; TSK-13-PT: Tampa Scale of Kinesiophobia-Portugese version; PCS-PT: Pain Catastrophizing Scale-Portuguese version.

## Data Availability

Not applicable.

## Abbreviations

CNSNP: Chronic Nonspecific Neck Pain; JPS; Joint Position Sense; DCF: Deep Cervical Flexors; STE: Specific Therapeutic Exercise; GPR: Global Postural Reeducation; CCFT: Cranio-Cervical Flexion Test; CROM: Cervical Range of Motion; PPT: Pressure Pain Threshold; CCF: Cranio-Cervical Flexors; NPRS: Numerical Pain Rating Scale; NDI-PT: Portuguese version of the Neck Disability Index; COP: Centre of Pressure; EMG: Electromyography; SCM: Sternocleidomastoid; AS: anterior scalene; TSK: Tampa Scale of Kinesiophobia; TSK-13-PT: Portuguese version of the 13-items Tampa Scale of Kinesiophobia; PCS: Pain Catastrophizing Scale; PCS-PT: Portuguese version of the PCS; ICC: intraclass correlation coefficient.
